# Catecholamine-induced Myocarditis in a Child with Pheochromocytoma

**DOI:** 10.4274/jcrpe.galenos.2019.2019.0045

**Published:** 2020-06-03

**Authors:** S. Ahmet Uçaktürk, Eda Mengen, Emine Azak, İbrahim İlker Çetin, Pınar Kocaay, Emrah Şenel

**Affiliations:** 1 Ankara City Hospital, Children’s Hospital, Clinic of Pediatric Endocrinology, Ankara, Turkey; 2 Ankara City Hospital, Children’s Hospital, Clinic of Pediatric Cardiology, Ankara, Turkey; 3 Ankara City Hospital, Children’s Hospital, Clinic of Pediatric Surgery, Ankara, Turkey

**Keywords:** Pheochromocytoma, myocarditis, neurofibromatosis type-1

## Abstract

Pheochromocytomas and paragangliomas (PPGLs) are rare neuroendocrine tumors. The clinical presentation of pediatric PPGLs is highly variable. In cases with pheochromocytoma (PCC), excess catecholamine may stimulate myocytes and cause structural changes, leading to life-threatening complications ranging from stress cardiomyopathy (CM) to dilated CM. Herein, we report the case of catecholamine-induced myocarditis in a child with asymptomatic PCC. A 12-year-and-2-month-old male patient with a known diagnosis of type-1 neurofibromatosis was brought to the emergency department due to palpitations and vomiting. On physical examination, arterial blood pressure was 113/81 mmHg, pulse was 125/min, and body temperature was 36.5 °C. Laboratory tests showed a leucocyte count of 12.8x10^3^ μL/L and a serum C-reactive protein level of 1.1 mg/dL (Normal range: 0-0.5). Thyroid function tests were normal, while cardiac enzymes were elevated. Electrocardiogram revealed no pathological findings other than sinus tachycardia. The patient was diagnosed with and treated for myocarditis as echocardiography revealed a left ventricular ejection fraction of 48%. Viral and bacterial agents that may cause myocarditis were excluded via serological tests and blood cultures. Blood pressure, normal at the time of admission, was elevated (140/90 mmHg) on the 5^th^ day of hospitalization. Magnetic resonance imaging revealed a 41x46x45 mm solid adrenal mass. The diagnosis of PCC was confirmed by elevated urinary and plasma metanephrines. The patient underwent surgery. Histopathology of the excised mass was compatible with PCC. It should be kept in mind that, even if there are no signs and symptoms of catecholamine elevation, CM may be the first sign of PCC.

**What is already known on this topic?**

The clinical presentation of pediatric pheochromocytoma and paragangliomas (PPGLs) is highly variable. Classic symptoms of catecholamine excess include headaches, diaphoresis and palpitations which may be episodic in nature. The most common symptom in children is sustained hypertension. Excessive catecholamine may stimulate myocytes and cause structural changes, leading to life-threatening complications ranging from stress cardiomyopathy (CM) to dilated CM. Catecholamine-induced myocarditis is a rare clinical manifestation seen in adult patients with pheochromocytoma.

**What this study adds?**

To our knowledge, no pediatric case presenting with myocarditis has been reported in the literature. Our patient was diagnosed with myocarditis as the first symptom without the expected signs and symptoms due to catecholamine elevation. Even if there are no signs and symptoms of catecholamine elevation, functional PPGLs may lead to CM.

## Introduction

Pheochromocytomas (PCC) and paragangliomas (PPGLs) are rare neuroendocrine tumors. The prevalence of PPGLs among children with hypertension is 1.7% (1). A PCC is a catecholamine-producing paraganglioma (PGL) of adrenal medulla origin. PGLs are tumors originating from sympathetic or parasympathetic paraganglia. The average age at admission for pediatric PPGLs is 11-13 years, and they are more common in males with a ratio of 2:1 (2). The clinical presentation of pediatric PPGLs is highly variable. The classic symptoms of catecholamine excess include headaches, diaphoresis and palpitations, which may be episodic in nature. This triad of the disease is present in about 54% of patients (3). The most common symptom in children, present in 60-90% of cases, is sustained hypertension (2).

Features of PCC are summarized by the “Rule of 10s”: 10% is malignant, 10% is extra-adrenal, 10% is bilateral, and 10% is hereditary. However, up to 80.4% of PCC in children are hereditary (4). PPGLs may be a part of hereditary syndromes, such as multiple endocrine neoplasia type 2A or 2B, Von Hippel-Lindau syndrome and neurofibromatosis type 1 (NF1) (1,2).

In cases with PCC, excess catecholamine may stimulate myocytes and cause structural changes, leading to life-threatening complications ranging from stress cardiomyopathy (CM) to dilated CM. Catecholamine-induced myocarditis is an infrequent clinical manifestation seen in adult patients with PCC (5).

## Case Report

**Figure 1:**
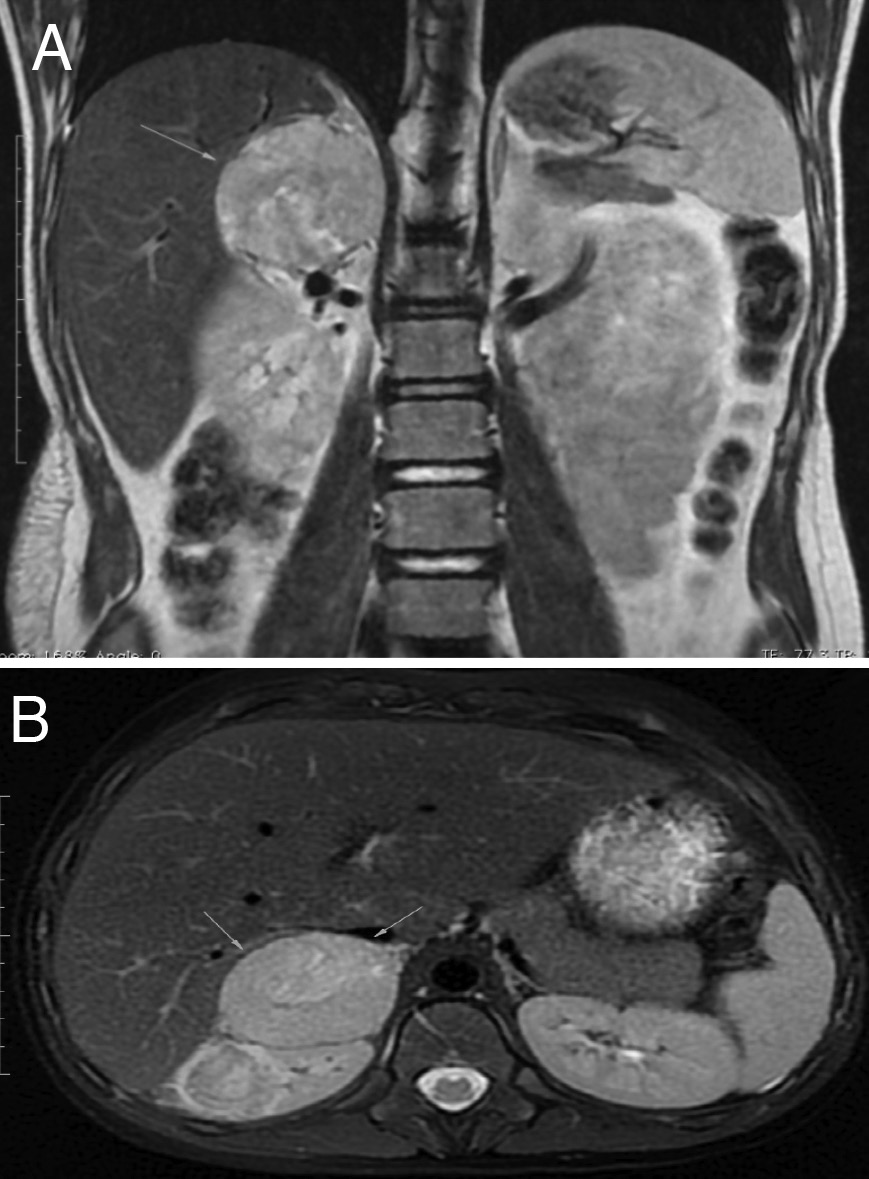
A, B) Abdominal magnetic resonance imaging findings: 41x46x45 mm solid mass lesion (green arrows), which had heterogeneous but diffuse contrast enhancement, was located between the liver and anterior upper pole of right kidney, displaced the liver to anterior

**Figure 2:**
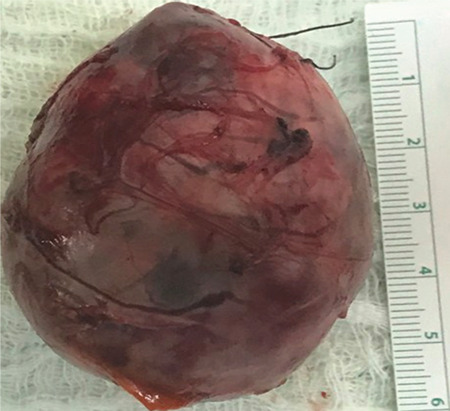
Excised mass: 6x5x4.5 cm nodular lesion

A 12-year-and-2-month-old male patient was brought to the emergency department due to palpitations that started at night together with repeated vomiting; ten episodes of vomiting were reported. It was learned that the patient had been followed up in the neurology clinic with the diagnosis of NF1, had no other complaint and did not use any medication. On physical examination, arterial blood pressure was 113/81 mmHg, pulse was 125/min, body temperature was 36.5 °C, weight was 30.5 kg (-1.86 standard deviation (SD)], and height was 137.9 cm (-1.87 SD). There were extensive cafe-au-lait spots, including in the lumbosacral and gluteal regions and covering the left thigh, and a 10x10 cm non-tender lumbar soft tissue lesion. The testicular volumes were 4/4 mL. There was no consanguinity between his parents. Laboratory tests showed a white blood count count of 12.8x10^3^ µL/L and a serum C-reactive protein concentration of 1.1 mg/dL [normal range (NR): 0-0.5]. Thyroid function tests were normal, while cardiac enzymes were elevated; troponin 1: 3.6 ng/mL (NR: 0-0.04), Pro-brain natriuretic peptide: 6730 ng/L (NR: 0-125), creatine kinase muscle B: 43 U/L (NR: 0-24). Electrocardiogram (ECG) revealed no pathological findings other than sinus tachycardia. The patient was diagnosed with and treated for myocarditis as echocardiography (ECHO) revealed a left ventricular ejection fraction (LVEF) of 48%, and mild mitral and aortic insufficiency. Serologic tests for the commonest viruses associated with CM, including Adenovirus, Coxsackie group B, Parvovirus, Herpes Simplex virus, Epstein-Barr virus, Rubella, and Human Immunodeficiency virus, and blood cultures were negative. Since blood pressure that was normal at the time of admission had become elevated (140/90 mmHg) by the 5^th^ day of hospitalization, the patient underwent Doppler ultrasound and subsequent abdominal magnetic resonance imaging (MRI). The MRI indicated that a 41x46x45 mm solid mass lesion, which had heterogeneous but diffuse contrast enhancement, was located between the liver and the anterior upper pole of the right kidney, displaced the liver to the anterior, and was heterogeneous hypointense in the T1A series and heterogeneous hyperintense in the T2A series (Figure 1A, 1B). Due to the combination of hypertension and an adrenal mass, a PCC was suspected and the relevant investigations were performed. 24-hr urine metanephrine was 13124 µg/L (NR: 50-250), 24-hr urine normetanephrine was 4987 ng/mL (NR: 84-422), plasma metanephrine was 136 ng/mL (NR: <90), adrenocorticotropic hormone was 21 pg/mL, and cortisol was 26 µg/dL. The diagnosis of PCC was confirmed by elevated levels of urinary and plasma metanephrines. Gallium-68-dodecanetetraacetic acid tyrosine-3-octreotate (^68^Ga-DOTATATE) positron emission tomography indicated a 40x55x45 mm mass with well-defined smooth margins between the upper pole of the right kidney and posteromedial of the right lobe of the liver. ACE inhibitor (Enalapril) and furosemide treatment initiated for the patient with the diagnosis of myocarditis were terminated. The patient was started on doxazosin treatment and subsequently on amlodipine for PCC. Doxazosin therapy was initiated at 1 mg/day, the dose was increased with blood pressure monitoring, and then, the calcium channel blocker amlodipine was added at 0.05 mg/kg/day. Blood pressure was brought under control (lowered below the 95^th^ percentile) with both drugs at 10 mg/day. The patient underwent surgery once the LVEF increased to 76%. A high-sodium diet was recommended before the surgery. A saline infusion was initiated the night before the surgery and continued during the surgery for volume expansion. Blood pressure monitoring was performed intraoperatively. No hypotension was observed during and after the excision of the mass. There was no complication during or after the surgery. Pathological findings of the excised mass were compatible with PCC (Figure 2). Histological and immunohistochemical analyses confirmed the diagnosis of PCC.

## Discussion

PCC-related CM is frequently associated with stress CM, such as ampulla or Takotsubo, in which there are ST segment changes on ECG and left ventricular apical ballooning. It has been reported with dilated and hypertrophic CM and more rarely with myocarditis in adult patients (5). To our knowledge, no pediatric case presenting with myocarditis has been reported in the literature. As catecholamine-related CM is reversible, early diagnosis and PCC resection are very important, and delayed diagnosis may lead to irreversible cardiac remodeling and death (5).

Our patient was diagnosed with myocarditis as the first symptom without the signs and symptoms typical of catecholamine elevation. The clinical presentation of functional PPGLs depends on differences in catecholamine secretion and release, as well as on individual patient sensitivities to catecholamines (6). Furthermore, patients with large tumors exhibit fewer symptoms because of metabolic degradation of most of the catecholamines produced leading to a clinical picture of relatively lower circulating free catecholamines but high urinary excretion of catecholamine metabolites (7). The large tumor diameter in our patient may be another factor in the absence of evident catecholamine-related symptoms.

At admission and during the early days of the first hospitalization, the patient’s normal blood pressure was attributed to the low ejection fraction due to myocarditis. Hypertension is reported in 65% of patients with PCC-related CM, and the classic triad of the disease (headache, palpitations, and diaphoresis) is reported in only 4%. The diagnosis of PCC-related CM is usually delayed due to atypical presentations in most of the patients (5).

Catecholamines create a positive inotropic effect by regulating cardiac functions at low concentrations but lead to the following harmful effects at high concentrations (8): epinephrine or norepinephrine activates protein kinase A by binding to B2 receptors and through cyclic adenosine monophosphate (cAMP) to produce an increased contractile response. Increased cAMP induces free radical formation, expression of stress hormone genes, and apoptosis. Excessive catecholamine levels cause functional hypoxia due to increased contractility, decreased blood flow due to coronary spasm, mitochondrial dysfunction caused by excess free fatty acids, and cardiomyocyte damage due to excess intracellular calcium. The catabolism of catecholamines proceeds by two major pathways regulated by monoamine oxidase and catechol-ortho-methyl transferase. When these enzymes become saturated and the concentration of circulating catecholamines is excessive, auto-oxidation mechanisms may be initiated, which leads to the formation of oxidized catecholamines (8).

Our patient had widespread cafe-au-lait spots and a plexiform neurofibroma; he had been followed up in the neurology department with the diagnosis of NF1. NF1 is an autosomal dominant disorder, which emerges as a result of *de novo* germline mutations in approximately half of the patients. The incidence of PCC among patients with NF1 has been reported to be between 2.9-14.6% (9,10). On the other hand, somatic NF1 mutations were detected in 25% of sporadic PPGLs (11). Considering the low prevalence and slow growth of PPGLs, it has been recommended that asymptomatic patients with NF1 should be screened every three years, starting from 10-14 years of age, and biochemical tests for PPGL should be performed before elective surgical procedures in patients with NF1 (9).

## Conclusion

In conclusion, even if there are no signs and symptoms of catecholamine elevation, PCC-related CM may arise. PPGLs should be considered during the evaluation of non-ischemic, non-valvular CM, even if there are no signs of catecholamine excess. Making an accurate diagnosis in the early period will protect these patients from life-threatening complications.
